# Supplementary material for: Digitally-enhanced lubricant evaluation scheme for hot stamping applications
Source: Nat Commun. 2022 Sep 30;13:5748. doi: 10.1038/s41467-022-33532-1 (PMC9525279; doi:10.1038/s41467-022-33532-1)
Supplement: Supplementary file 2 — Supplementary Information [file 41467_2022_33532_MOESM2_ESM.pdf]

Supplementary information

Digitally-enhanced lubricant evaluation scheme for hot stamping applications

Xiao Yang[1,2], Heli Liu[1,2], Saksham Dhawan[1,2], Denis J. Politis[3], Jie Zhang[1], Daniele Dini[1], Lan Hu[1,2], Mohammad M. Gharbi[4], Liliang Wang[1,2*].

[1] Department of Mechanical Engineering, Imperial College London, London, SW7 2AZ, UK

[2] SmartForming Research Base, Imperial College London, London, SW7 2AZ, UK

[3] Department of Mechanical and Manufacturing Engineering, University of Cyprus, Nicosia, 1678, Cyprus

[4] Houghton Deutschland GmbH, Giselherstraße 57, 44319 Dortmund, Germany

[*] Corresponding author.

E-mail address: Liliang.wang@imperial.ac.uk (Liliang Wang).

Supplementary Figures 1-8

Supplementary Tables 1-5

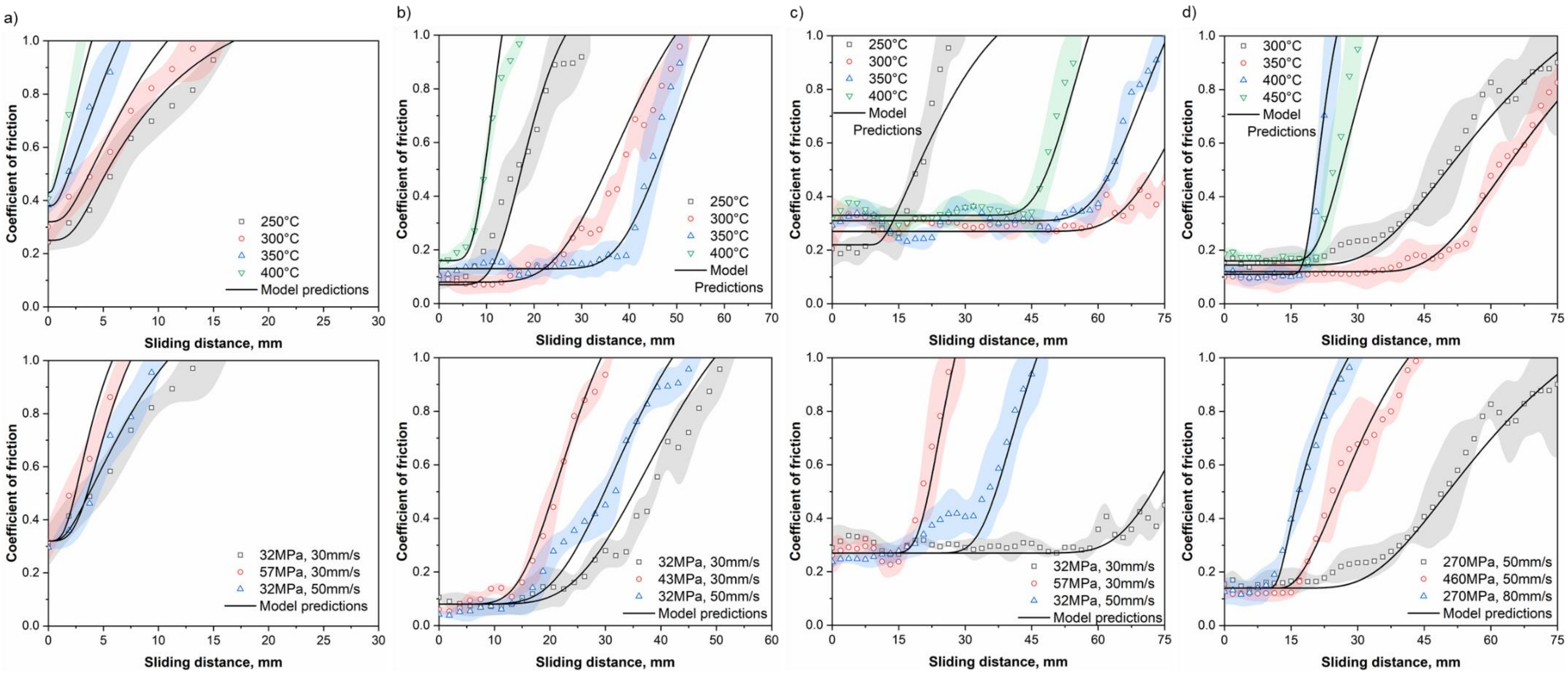

Testing conditions		
Temperature (ºC)	Pressure (MPa)	Speed (mm/s)
250	80	30
300	32	30
350	19	30
400	12	30
300	57	30
300	32	50

Testing conditions		
Temperature (ºC)	Pressure (MPa)	Speed (mm/s)
250	80	30
300	32	30
350	19	30
400	12	30
300	43	30
300	32	50

Testing conditions		
Temperature (ºC)	Pressure (MPa)	Speed (mm/s)
250	80	30
300	32	30
350	19	30
400	12	30
300	57	30
300	32	50

Testing conditions		
Temperature (ºC)	Pressure (MPa)	Speed (mm/s)
300	270	50
350	220	30
400	180	50
450	85	30
300	460	50
300	270	80

Supplementary Fig. 1 | Comparisons between model predictions and experimental results of the COF evolution under constant contact conditions for four lubricant candidates, and the corresponding testing conditions. a, Lubricant #1. **b**, Lubricant #2. **c**, Lubricant #3. **d,** Lubricant #4. Under different temperatures, contact pressures, and sliding speeds. Solid lines, modelling results; scatters, average values; envelopes, standard deviations.

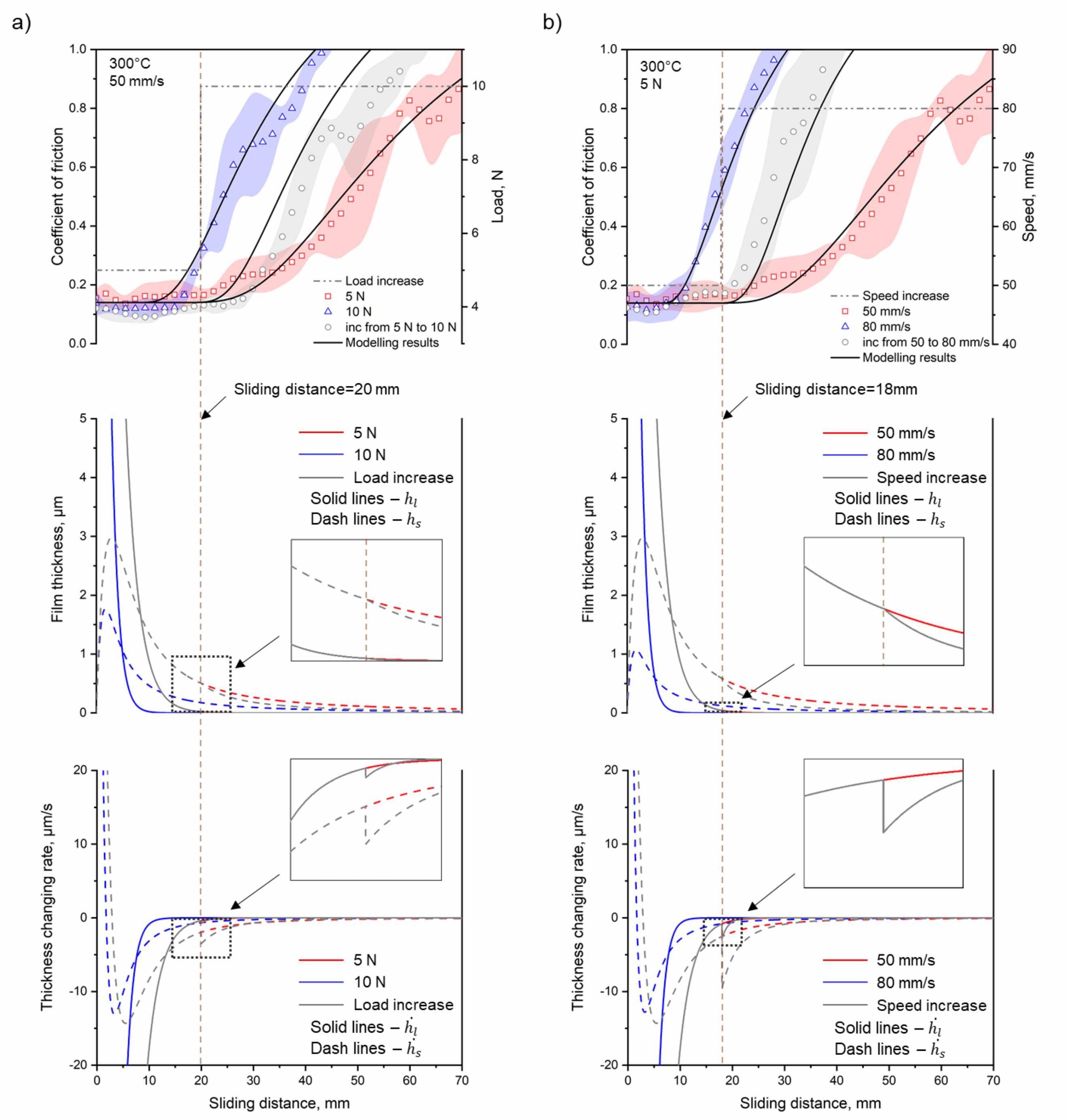

Supplementary Fig. 2 | Model responses in evolutions of film thickness and thickness changing rate for the two-phase lubricant under complex loading conditions. a) load increase test; b) speed increase test. In the load increase test, there was an abrupt change of contact load from 5 N to 10 N at the sliding distance of 20 mm. Due to this abrupt change, the thickness changing rate of both liquid lubricant film and solid tribo-layer also experienced a sudden drop accordingly, leading to deviations of film thickness away from the constant loading curve and, thus, earlier breakdown was observed in the load increase test as was successfully captured and predicted by the calibrated interactive friction model. Similar phenomena could also be found under the changes of speed and temperature. According to this advanced and inherent feature of the friction model, the COF evolution could be determined under complex contact conditions, with good agreements, even though the model parameters were calibrated against testing results under constant contact conditions.

a)

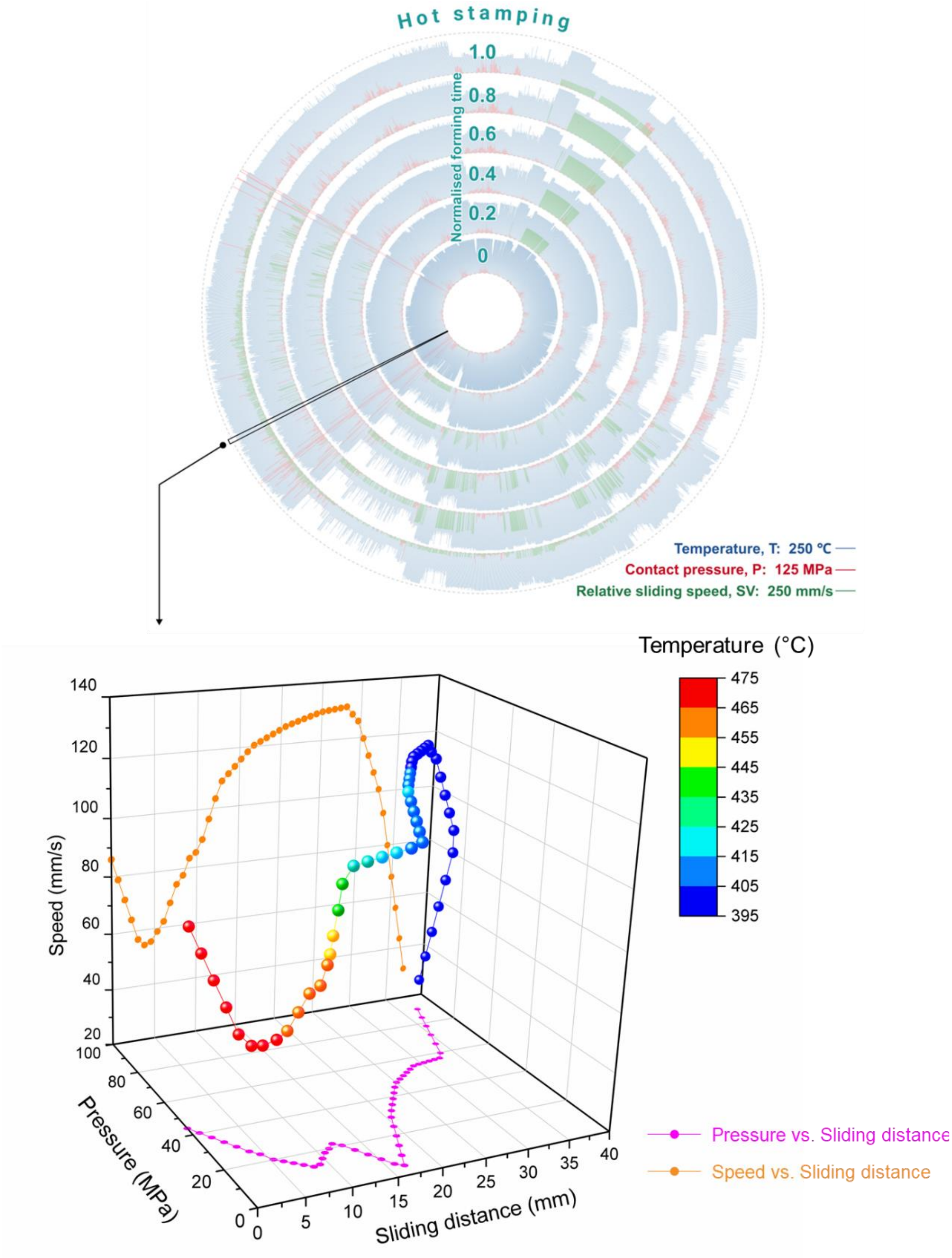

Hot stamping
1.0
0.8
0.6
0.4
0.2
0
Normalised forming time
Temperature, T: 250 °C
Contact pressure, P: 125 MPa
Relative sliding speed, SV: 250 mm/s
Temperature (°C)
475
465
455
445
435
425
415
405
395
Speed (mm/s)
Pressure (MPa)
Sliding distance (mm)
Pressure vs. Sliding distance
Speed vs. Sliding distance

b) c)

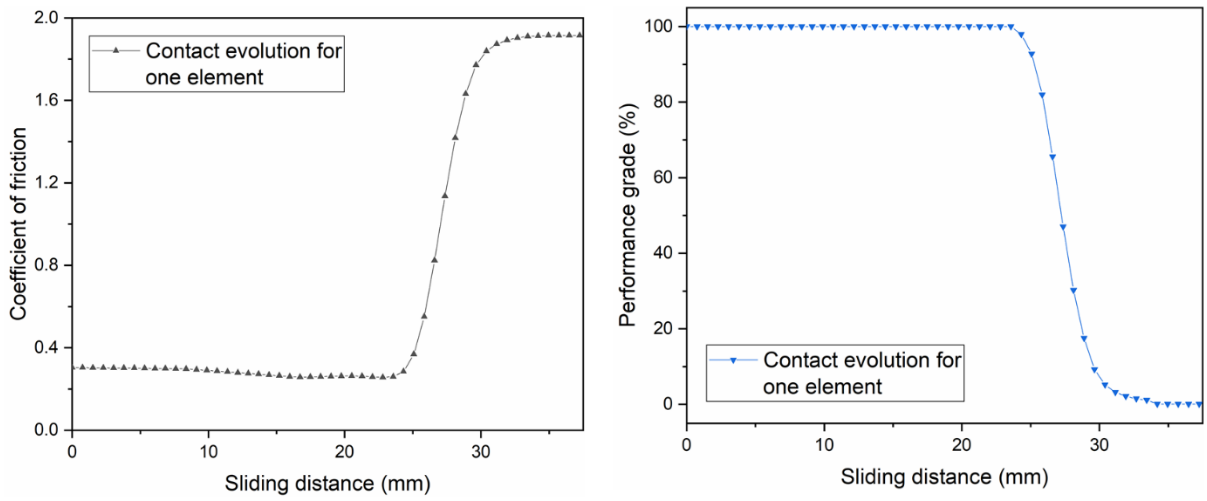

Supplementary Fig. 3 | Example of contact condition evolution history for an individual element at the tool-workpiece interface and prediction of COF evolution and corresponding performance grade evolution based on the interactive friction model. a, Example evolution history of contact pressure, sliding speed and interfacial temperature as a function of the sliding distance for one element extracted from hot stamping. The radial bar chart plots the contact condition (T, P, SV) evolutions of each tool element as a function of the normalised forming time along the radial direction and each radius represents an individual tool element. **b**, Application of the interactive friction model for the prediction of COF evolution following the evolution history demonstrated in **a**. **c,** Performance grade evolution transformed from COF evolution following the evolution history demonstrated in **a**.

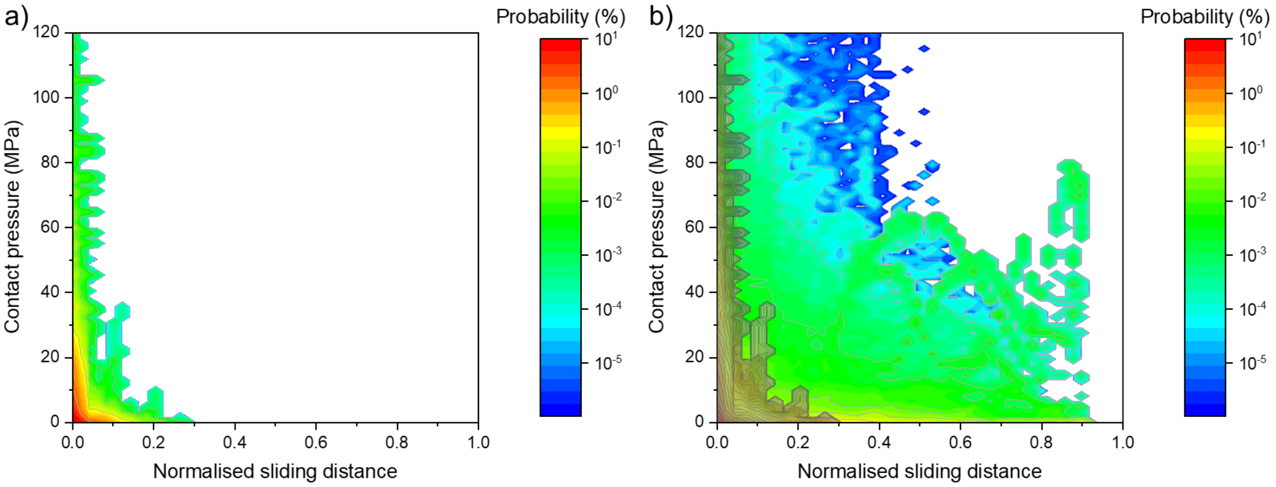

Supplementary Fig. 4 | Further experimental validation of the LLD predictions by performing hot stamping of an automotive component. a) DC of the hot stamping process of the component, b) Comparisons between DC of the component and overall hot stamped components. Forming of this component experiences relatively short normalised sliding distances of generally less than 0.3.

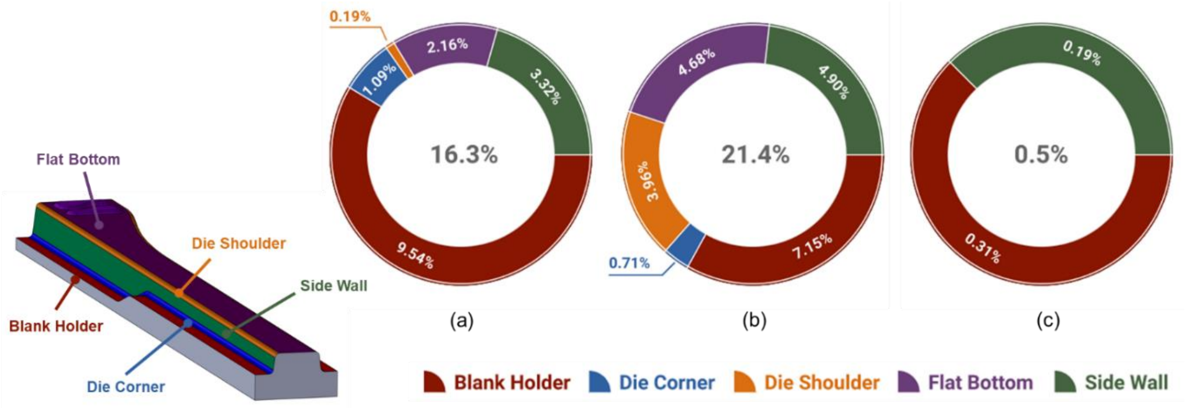

Supplementary Fig. 5 | Identification of the most suitable lubricant candidate for this experimental validation and data distribution analysis of the lubricant failure region during the forming process of the component. a) lubricant #1, b) lubricant #2 and c) lubricant #3. The dangerous region for this forming test was the blank holder and side wall areas.

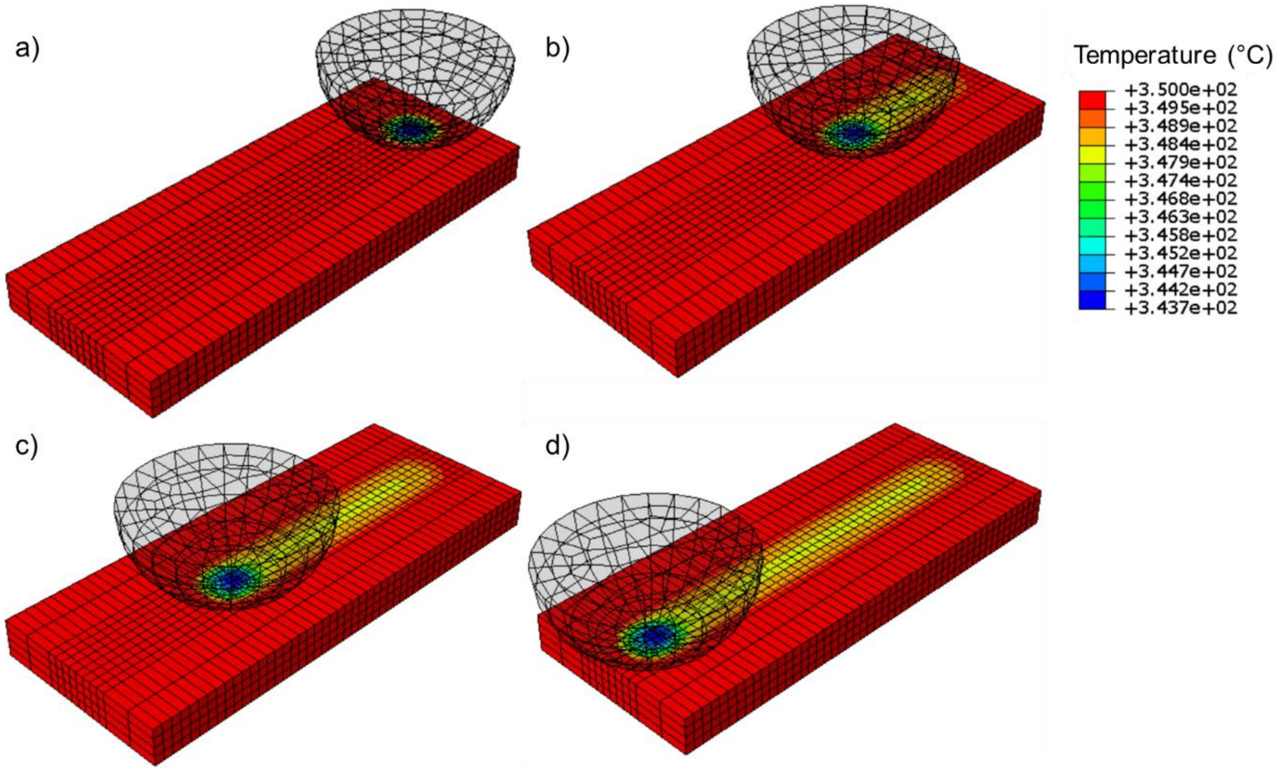

Supplementary Fig. 6 | Interfacial temperature distribution across the sliding wear track investigated by using Abaqus. Under the initial blank temperature of 350°C, relative sliding speed of 30 mm/s and contact pressure of 19 MPa, as a function of the sliding time: (a) 0s, (b) 0.05s, (c) 0.1s and (d) 0.15s. Frictional heat generation due to relative sliding, conductance between the hot aluminium blank and cold steel pin and convection to the ambient air were considered in this temperature distribution analysis. Radiation was negligible due to the relatively low emissivity coefficient of aluminium. The interfacial heat transfer coefficient (IHTC) between AA7075 aluminium alloy and tool steel indicates a stable value of IHTC as the contact pressure is greater than 10 MPa. A constant temperature boundary condition is assumed at the central plane of the blank, where thermos-couples were located, due to the insulation of thermal box and short sliding time.

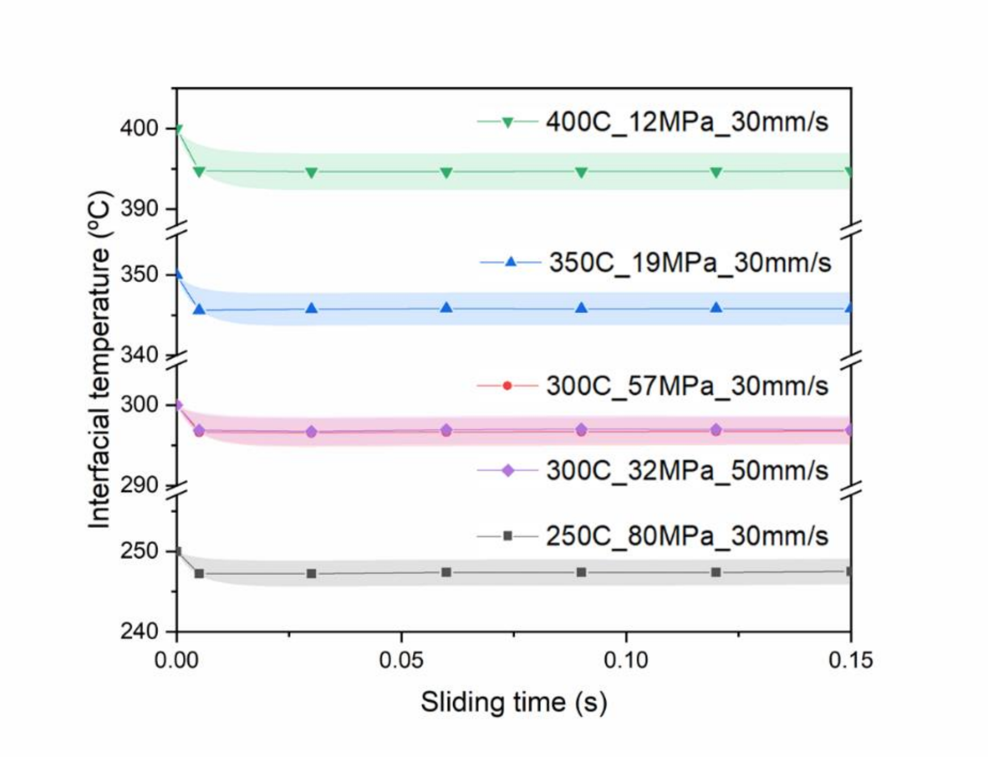

Supplementary Fig. 7 | Simulation results of the actual interfacial temperature under different testing conditions. Scatter represents the average value; envelope represents the standard deviation. The temperature deviation between the contact interface and the measure point is less than 1.3% of the nominated value, which is acceptable and presents minor effects on the final lubricant evaluation results. This can be expected due to the thin aluminium blank specimen (1.6 mm) and the small contact area between the pin and blank (~0.78 mm^2), which would generate limited frictional heat during sliding.

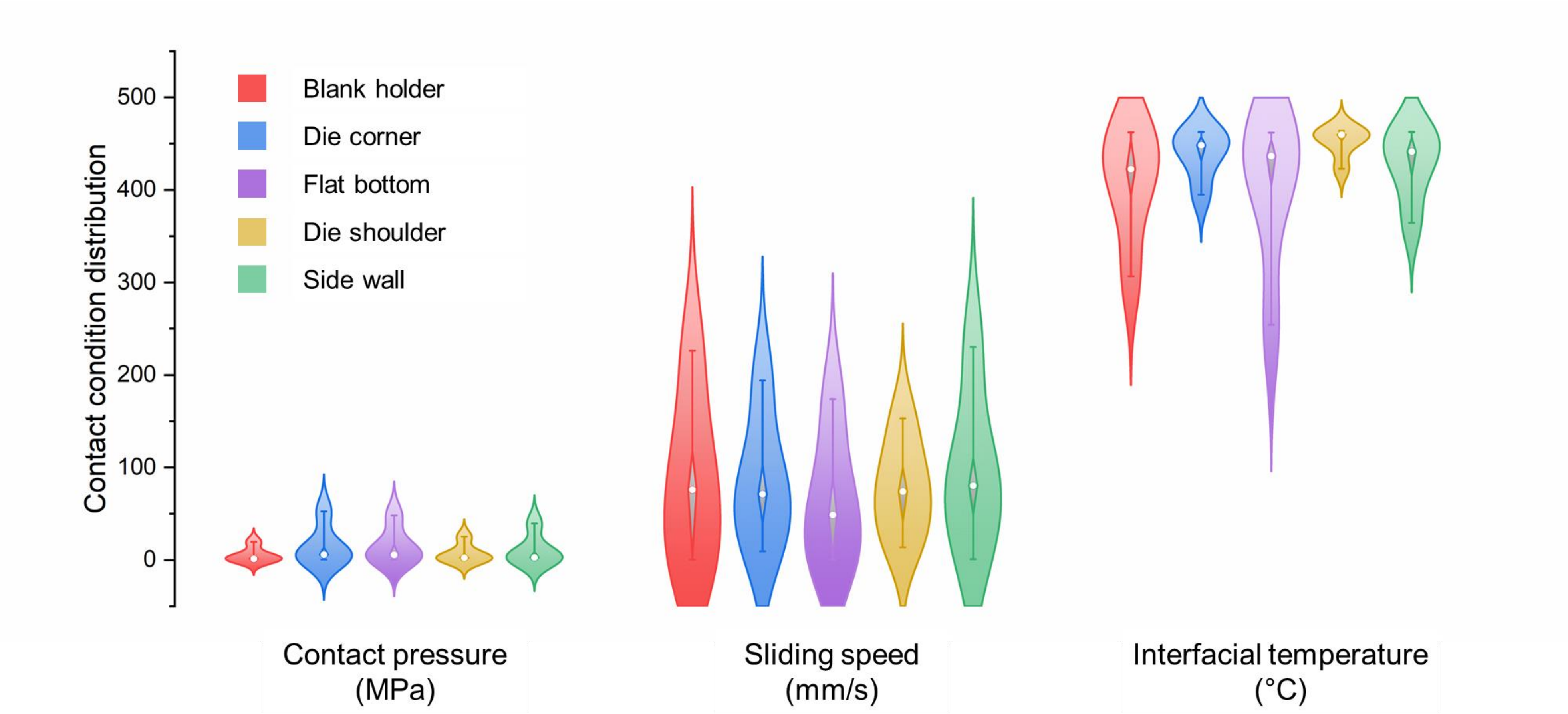

Supplementary Fig. 8 | Contact condition distribution of the individual forming region (blank holder, die corner, flat bottom, die shoulder and side wall region) in the hot stamping process. Contact pressure distribution, sliding speed distribution and interfacial temperature distribution are shown. The white circle in the middle represents the medium, the diamond box indicates the interquartile range, and the caps indicates the 5% and 95% range, respectively.

Supplementary Table 1. Information of 4 lubricants evaluated in this study

Lubricant	Type	Appearance	Specific gravity @ 20°C	Kinematic viscosity (cSt) @ 20°C	Dry matters (%)
#1	Water-based	Clear liquid	1.2	1167 (ASTM D789)	37
#2	Water-based	Clear liquid	0.99	19 (ASTM D789)	--
#3	Water-based	Clear liquid	1.17	256 (ASTM D789)	32
#4	Oil-graphite based	Black	1.32	131 (base fluid) (ASTM D445)	25

Supplementary Table 2. Model parameters of the interactive friction model for lubricant #1

Parameter	$\lambda_1(\mu m)$	$\lambda_2(-)$	$k_1(-)$	$k_2(-)$	$k_\alpha(-)$	$k_s(-)$
Value	0.89	0.81	1.20	2.01	1.02	1.22
Parameter	$D_0(s^{-1})$	$Q_D(kJ \cdot mol^{-1})$	$c[mm(GPa \cdot s)^{-1}]$		$R[J(K \cdot mol)^{-1}]$	$m(-)$
Value	4.98e+4	35.01	701.65		8.314	0.37
Parameter	$K_0(s^{-1})$	$Q_K(kJ \cdot mol^{-1})$	$n_P(-)$	$n_v(-)$		
Value	5.03e+4	60.11	1.19	1.99		

Supplementary Table 3. Model parameters of the interactive friction model for lubricant #2

Parameter	$\lambda_1\ (\mu m^{-1})$	$\lambda_2(-)$	$c[mm(GPa \cdot s)^{-1}]$	$k_p(-)$	$k_v(-)$
Value	35	0.45	17.45	1.93	1.32
Parameter	$k_\eta(-)$	$\eta_0\ (mm^2/s)$	$Q_\eta\ (kJ \cdot mol^{-1})$	$R[J(K \cdot mol)^{-1}]$	
Value	2.96	0.024	16.29	8.314	

Supplementary Table 4. Model parameters of the interactive friction model for lubricant #3

Parameter	$\lambda_1(\mu m)$	$\lambda_2(-)$	$k_1(-)$	$k_2(-)$	$k_\alpha(-)$	$k_s(-)$
Value	0.83	4.20	1.73	1.52	1.11	0.88
Parameter	$D_0(s^{-1})$	$Q_D(kJ \cdot mol^{-1})$	$c[mm(GPa \cdot s)^{-1}]$		$R[J(K \cdot mol)^{-1}]$	$m(-)$
Value	4.47e+4	37.91	3.32e+4		8.314	0.32
Parameter	$K_0(s^{-1})$	$Q_K(kJ \cdot mol^{-1})$	$n_P(-)$	$n_v(-)$		
Value	4.80e+4	74.36	1.84	2.13		

Supplementary Table 5. Model parameters of the interactive friction model for lubricant #4

Parameter	$\lambda_1(\mu m)$	$\lambda_2(-)$	$k_1(-)$	$k_2(-)$	$k_\alpha(-)$	$k_s(-)$
Value	0.28	2.21	1.25	2.59	1.17	1.78
Parameter	$D_0(s^{-1})$	$Q_D(kJ \cdot mol^{-1})$	$c[mm(GPa \cdot s)^{-1}]$		$R[J(K \cdot mol)^{-1}]$	$m(-)$
Value	1.19e+5	47.63	1.04		8.314	0.25
Parameter	$K_0(s^{-1})$	$Q_K(kJ \cdot mol^{-1})$	$n_P(-)$	$n_v(-)$		
Value	8.77e+3	70.48	1.09	2.83		
